# *KIT* Mutations Correlate with Higher Galectin Levels and Brain Metastasis in Breast and Non-Small Cell Lung Cancer

**DOI:** 10.3390/cancers14112781

**Published:** 2022-06-03

**Authors:** Avery T. Funkhouser, Alexander M. Strigenz, Bailey B. Blair, Andrew P. Miller, Jonah C. Shealy, Joseph A. Ewing, Julie C. Martin, Christopher R. Funk, William J. Edenfield, Anna V. Blenda

**Affiliations:** 1 Department of Biomedical Sciences, University of South Carolina School of Medicine Greenville, Greenville, SC 29605, USA; averytf@email.sc.edu (A.T.F.); strigenz@email.sc.edu (A.M.S.); bergmanb@email.sc.edu (B.B.B.); apm7@email.sc.edu (A.P.M.); jcshealy@email.sc.edu (J.C.S.); 2 Data Support Core, Prisma Health, Greenville, SC 29605, USA; alex.ewing@prismahealth.org; 3 Prisma Health Cancer Institute, Prisma Health, Greenville, SC 29605, USA; julie.martin@prismahealth.org (J.C.M.); jeffery.edenfield@prismahealth.org (W.J.E.); 4 Department of Hematology and Medical Oncology, Emory University School of Medicine, Atlanta, GA 30322, USA; ronnie.funk@emory.edu

**Keywords:** galectin, *KIT* gene, mutation, c-Kit, CD117, breast cancer, non-small cell lung cancer, ELISA, cancer hotspot panel

## Abstract

**Simple Summary:**

Galectins are a family of β-galactoside binding proteins whose levels are altered in various stages of different types of cancer. This study provides an analytical comparison of 50 frequently mutated genes in two common cancers and the serum levels of the galectin proteins. The goal is the revelation of potential relationships between the mutation status of these genes and serum levels of galectins. We found that mutations in the *KIT* gene (which codes for the proto-oncogene c-KIT protein) are associated with increased circulating levels of certain galectins. We also found that patient samples originating from brain tissue have a higher likelihood of having a mutation in the *KIT* gene. Understanding the relationship between cancer-critical gene mutations and serum galectin levels could provide a feasible and non-invasive avenue to better understand the tumor’s unique genetic profile.

**Abstract:**

To investigate a potential role for galectins as biomarkers that enable diagnosis or prognostication of breast or non-small cell lung cancer, the serum levels of galectins -1, -3, -7, -8, and -9 of cancer patients determined by ELISA assays were compared to the mutation status of 50 known cancer-critical genes, which were determined using multiplex PCR in tumors of the same patients. Mutations in the *KIT* proto-oncogene, which codes for the c-Kit protein, a receptor tyrosine kinase, correlated with higher levels of galectins -1, -3, -8, and -9 in breast cancer patients and galectin-1 in non-small cell lung cancer patients. Mutations in the *KIT* gene were more likely found in brain metastases from both of these primary cancers. The most common *KIT* mutation in our panel was p.M541L, a missense mutation in the transmembrane domain of the c-Kit protein. These results demonstrate an association between *KIT* oncogenic signaling and elevated serum galectins in patients with metastatic disease. Changes in protein trafficking and the glycocalyx composition of cancer cells may explain the observed alterations in galectin expression. This study can be useful for the targeted selection of receptor tyrosine kinase and galectin inhibitor anti-cancer treatments.

## 1. Introduction

Lung cancer has one of the lowest 5-year survival rates of any cancer in the United States at 21% and is the greatest cause of cancer deaths in both men and women. Although the 5-year relative survival rate of breast cancer is 90% for all subtypes, its high incidence still results in tens of thousands of deaths annually in the United States [1]. Once metastasis occurs, the survival rate greatly drops, with a majority (66.7%) of all solid tumor cancer deaths being caused by metastasis [2]. Sixteen to twenty percent of those diagnosed with lung cancer, and 5.1% with breast cancer, develop metastasis to the brain [3,4]. The high incidence and metastatic ability make these cancers of primary interest for study.

### 1.1. Cancer-Critical Genes

Multiple genes in the human genome, when mutated, enable the development and progression of neoplastic tissue. Oncogenes are genes that, when mutated, create a product with a gain-of-function (GOF) that allows it to contribute to the dysregulation of the cell. Conversely, tumor suppressor genes lose their ability to protect the cell from dysregulated growth and proliferation when they are mutated. This paper refers to both groups of genes collectively as “cancer critical” genes.

**Figure 1:**
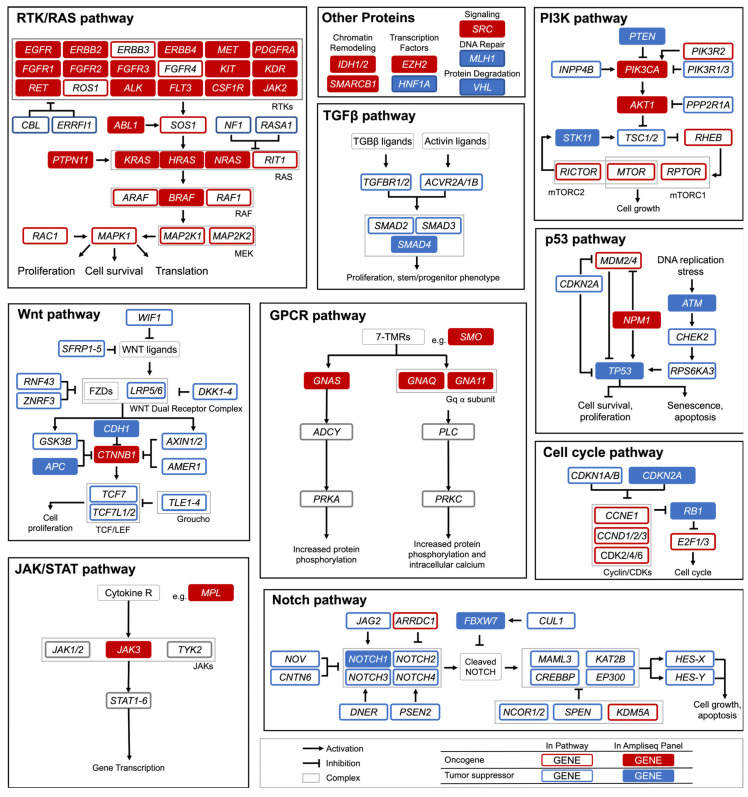
Signaling Pathways with Cancer Critical Genes. Hotspot panel genes in this study are shaded in red (oncogenes), and blue (tumor suppressor genes). RTK: Receptor Tyrosine Kinase; RAS: Rat Sarcoma; TGFβ: Transforming Growth Factor Beta; PI3K: Phosphoinositide 3-kinase; GPCR: G-Protein Coupled Receptor; JAK: Janus Kinase; STAT: Signal Transducer and Activator of Transcription Proteins. Figure modified from Sanchez-Vega et al. [6].

A recent comprehensive study of 9423 tumor exomes identified 299 cancer driver genes [5]. This study evaluates 50 of those cancer-critical genes, providing a broad screening of genes commonly mutated in multiple cellular pathways. The nine involved pathways are the RTK/RAS/MAP, TGFβ, PI3K, Wnt, GPCR, p53, JAK/STAT, Notch, and the cell cycle pathway. These genes and their respective pathways are highlighted in Figure 1. The graphic is not comprehensive of all the possible cancer-critical genes or the full signaling pathways, it is instead designed to highlight the genes used in this study and their potential contribution to unregulated proliferation.

Seven genes were not a part of any canonical cancer-causing pathway or were members of multiple pathways (e.g., *SRC*); these are grouped together as “Other” in Figure 1. These genes are involved in DNA repair, genomic stability, epigenetic modification, etc.

The *KIT* gene codes for c-Kit, a class III receptor tyrosine kinase (RTK) which binds to extracellular Stem Cell Factor (SCF) and activates the PI3K, JAK/STAT, and MAPK pathways in hematopoietic cells, resulting in proliferation and differentiation [7,8,9]. This RTK is also highly expressed by glandular and myoepithelial breast cells [10]. Furthermore, c-Kit is known to play roles in several cancers via gain-of-function and loss-of-function mutations; most notably in gastrointestinal stromal tumors (GIST), and also in melanoma and thyroid carcinoma [11,12,13,14]. Several mutations in c-KIT have been associated with the development of cancers. These mutations are typically missense mutations that usually result in gain-of-function or an otherwise unknown result [15].

The incidence of brain metastasis in breast cancer patients is possibly increasing and is high in patients with hormone receptor-negative tumors with human epidermal growth factor receptor 2 (HER2) overexpression [16,17,18,19,20,21]. Similarly, patients with specific molecular subtypes of NSCLC have an increased predisposition for brain metastases, such as those with an epidermal growth factor receptor (EGFR) mutation, or anaplastic lymphoma kinase (ALK) rearrangement [22,23,24].

Targeted cancer therapy offers more precise cancer treatment with fewer cytotoxic effects on non-cancer cells [25]. This level of therapy requires knowing the cancer’s specific genetic makeup to identify druggable targets. For example, osimertinib, a tyrosine kinase inhibitor, is a targeted therapy for patients with NSCLC with specific sensitizing mutations (p.Thr790Met and p.Leu858Arg) in the *EGFR* gene [26,27,28,29,30,31,32]. A more thorough understanding of the cellular biology of cancer will reveal the therapeutic targets involved in growth and is a promising strategy for reducing mortality from cancer and its metastases.

### 1.2. Galectins and Their Role in Cancer

Galectins (formerly known as S-type lectins) are a family of lectin proteins which share a domain with high-affinity binding for β-galactoside sugars. Galectins are divided into three subfamilies based on their structures: prototypical, chimeric, and tandem-repeat [33]. Among other functions, galectins are players in the innate immune system, triggering immune responses as well as resolving inflammation [34]. Further, galectins modulate adaptive immune responses, such as Gal-9 or Gal-1, acting to dampen activated T cell responses [35]. Galectins also have several functions outside of the immune system. They interact with cellular proteins via binding to protein glycosylation sites [36,37]. They can form lattice networks with cell membrane receptors and modulate the functions and transportation of the receptors [38]. Galectins have intracellular interactions as well and enhance oncogenic signals and promote tumor proliferation [39].

Galectin-1, -3, and -9 have been implicated in cancer progression, metastasis, and angiogenesis [40,41,42,43,44]. Galectins-7 and -8 have less studied properties in cancer but are known to support metastatic breast cancer and colon cancer, respectively [45,46]. Their understudied status warrants their inclusion in this study.

In breast cancer, galectins have several roles. Galectins-1 and -3 specifically have been implicated in the progression of lesions into metastatic disease through their roles in cell-to-cell and cell-to-extracellular matrix interactions [47]. Galectin-7 has been found to have interactions with p53 that can induce chemoresistance [45,48]. Increased levels of galectin-8 were shown to lead to lower survival rates [49]. Lastly, galectin-9 has increased expression in breast cancer, and its interactions with Tim-3 may provide an escape from cytotoxic T cells [50,51].

In regard to NSCLC, galectin-1 is overexpressed in these cell lines and in tissue samples from lung cancer patients [52,53]. Knockdown of Gal-1 in lung adenocarcinoma results in reduced tumor growth in vivo and inhibited migration, invasion, and colony formation in vitro [53]. Galectin-3 is also more highly expressed in NSCLC and augments tumorigenesis, invasion, metastasis, and tumor immunity [54]. Galectins -7 and -8 have been shown to have higher mRNA expression levels in NSCLC [55]; however, no studies have examined their roles. Galectin-9 expression in NSCLC is found to be a favorable prognostic marker due to interactions between tumor-infiltrating lymphocytes and tumor cells [55,56].

The tissue levels of galectins are well known to be altered in breast and lung cancer [42,52,57,58,59,60,61,62]. This results in measurably altered serum levels of these galectins [52,63,64,65]. The mechanism for the altered serum expression of galectins in cancer patients remains unclear, however, there are potential explanations. Galectins are secreted in a currently enigmatic non-classical pathway and their trafficking is controlled at different points within the cell [66,67,68,69,70]. The abnormal cellular processes of cancer cells quite possibly result in the dysregulation of the processes involved in galectin secretion. Additionally, while normal cellular glycosylation is required for proper functioning, cancer cells have deviant glycosylation [71,72,73]. This could disturb the type and number of glycoconjugates for which galectins bind. The alterations in both trafficking and the glycomic profile, in turn, could lead to altered galectin levels in these neoplastic tissues, resulting in their demonstrably different serum levels.

As such, levels of galectin-3 have been especially implicated as an emerging biomarker for neoplasms [39,44,74,75,76,77]. Other galectins also have the potential to serve as biomarkers for diseases, such as cancer [77,78,79,80].

Given galectin’s dysregulation in the cancer environment and a possible therapeutic target, several galectin inhibitors have been developed [41,78,81,82]. GR-MD-02 (a galectin-3 inhibitor) is currently in clinical trials to evaluate its usefulness in cancer patients with melanoma, NSCLC, and squamous cell head and neck cancer (NCT02117362, NCT02575404).

Galectins interact with proteins commonly mutated in cancer. Galectin-1 and -3 have interactions with the RAS family of proteins [53,83,84,85,86] and EGFR and TGFβ receptors [87]. Thus, the interplay between galectin levels and mutations in these cancer-critical genes has clinical importance.

Finally, galectins are known players in cancer metastasis. Galectins -1 and -3 are particularly well studied in this aspect. Galectin-3 has been identified as a metastasis-related protein as early as 1998 [88]. Galectin-1 is upregulated in more advanced breast cancers of higher TNM stages and correlates with metastasis to regional lymph nodes [58,89]. Molecularly, the lectin interacts with laminin and fibronectin to promote aggregation [90,91]. Galectin-1 is able to upregulate MMP-2 and MMP-9 and reorganize cytoskeletal elements by activating Cdc42 to increase the amount of filopodia in oral squamous cell carcinoma cells [92]. Knockdown of galectin-1 reduced prostate cancer migration by suppressing androgen receptors and Akt signaling [93].

While many studies show changes in galectin levels during cancer, no comprehensive work has been done to correlate galectin levels with cancer-critical gene mutations in cancer patients [65,77,78,80,94,95]. This paper seeks to provide an initial exploration into serum galectin levels and their correlation with cancer-critical gene mutations in breast and non-small cell lung cancer patients.

## 2. Materials and Methods

### 2.1. Patient Samples

Seventy-four cancer patient serum samples were obtained from the Prisma Health Cancer Institute (PHCI) biorepository (Greenville, SC, USA). The collection years ranged from 2012–2018. The PHCI biorepository houses inventory, including live cryopreserved, snap-frozen, and formalin-fixed paraffin-embedded tissues, and blood (whole blood, plasma, and serum). Patient donor permission was obtained via participant informed consent prior to the collection and storage of specimens. The biorepository standard operating procedures include specimen handling and tracking (i.e., collection, processing, storage) and facilities management and operations (i.e., equipment maintenance and monitoring). The PHCI biorepository has been acknowledged in various publications, having provided all specimen types in its inventory for previously conducted research projects [65,96,97].

Thirty-five samples were from breast cancer patients (F:M 1:0, median age 60.6, min-max 33–87) and the other 39 were from NSCLC patients (F:M 17:22, median age 65.1, min-max 47–79). Ten samples were obtained of stages I, II, and III of breast and lung cancer, 5 of stage IV breast cancer, and 9 of stage IV lung cancer. In the breast cancer samples, 31 were ductal, 2 were lobular and 2 were coded non-specifically as “adenocarcinoma” histology. In the lung cancer samples, 24 were adenocarcinoma, 13 were squamous cell and 2 were large cell histology. The samples from the patients contained a random mix of primary tumors and metastatic tissue.

Patient information was collected from the PHCI database. The information included demographic data, such as age, race, gender, and smoking status, as well as tumor data, including TNM staging, grade, histology, site, and cancer stage. This information is available in Supplemental Materials (Tables S1 and S2)..

### 2.2. Galectin Profiling

The patient’s serum was used to determine the circulating galectin levels using an enzyme-linked immunosorbent assay (ELISA) [65]. This study used a subset of the data described by Blair et. al. (2021). Galectin-1, -3, and -9 concentrations were obtained using the ELISA kits from R&D Systems (Minneapolis, MN, USA). Galectin-7 and -8 concentrations were determined using the ELISA kits from Invitrogen (Carlsbad, CA, USA). Each sample was assayed four times. ELISA kit quality control information can be found in Supplemental Materials as Table S3. 

### 2.3. Cancer HotSpot Panel

Mutation information of the patient’s tumors was provided by the PHCI. The cancer hotspot panel screening was performed by Precision Genetics (Greenville, SC, USA). The Ion Ampliseq Cancer Hotspot Panel, v2 (Life Technologies Corporation, Carlsbad, CA, USA), was used to determine the mutation status of 50 genes in the patients. This panel amplifies 207 amplicons, which cover approximately 2800 mutations in the Catalogue of Somatic Mutations in Cancer (COSMIC) from 50 oncogenes and tumor suppressor genes.

Oncogenes: *ABL1*, *AKT1*, *ALK*, *BRAF*, *CSF1R*, *CTNNB1*, *EGFR*, *ERBB2*, *ERBB4*, *EZH2*, *FGFR1*, *FGFR2*, *FGFR3*, *FLT3*, *GNA11*, *GNAQ*, *GNAS*, *HRAS*, *IDH1*, *IDH2*, *JAK2*, *JAK3*, *KDR*, *KIT*, *KRAS*, *MET*, *MPL*, *NPM1*, *NRAS*, *PDGFRA*, *PIK3CA*, *PTPN11*, *RET*, *SMARCB1*, *SMO*, *SRC*.

Tumor Suppressors: *APC*, *ATM*, *CDH1*, *CDKN2A*, *FBXW7*, *HNF1A*, *MLH1*, *NOTCH1*, *PTEN*, *RB1*, *SMAD4*, *STK11*, *TP53*, *VHL*.

The AmpliSeq Cancer Hotspot panel (v2), from which all variants were identified, was validated as a laboratory-developed test (LDT) under the Clinical Laboratory Improvement Amendments (CLIA) guidelines. The accuracy of all variant calls was validated at 99.8%. The sensitivity of the variants was detected at a lower limit of 5% allele frequency down to 30% tumor content (cell admixture). The precision of variant detection was shown to be 99.8% between operators and 98.9% within the operator. False variant calls, a measure of specificity, were less than 1% from the CLIA validation.

Each sequencing run had minimum criteria for variant calls. Coverage across the entire panel must be greater than 90% at 300X for the sequencing run to be analyzed further. A minimum read depth of 100X and 5% allele frequency must be observed for individual variants to be reported. Finally, homopolymer indels and variants within 10 bp of amplicon ends were filtered to minimize the likelihood of false positives.

Each sequencing run included the AcroMetrix Oncology Hotspot Control, which is designed to control the hundreds of amplicons targeted by next-generation sequencing (NGS) panels. It contains over 500 mutations from the COSMIC database and has five variant types of varying nucleotide lengths. The 53 genes represented in the AcroMetrix Oncology Hotspot Control are: *ABL1*, *AKT1*, *ALK*, *APC*, *ATM*, *BRAF*, *CDH1*, *CDKN2A*, *CSF1R*, *CTNNB1*, *EGFR*, *ERBB2*, *ERBB4*, *EZH2*, *FBXW7*, *FGFR1*, *FGFR2*, *FGFR3*, *FLT3*, *FOXL2*, *GNA11*, *GNAQ*, *GNAS*, *HNF1A*, *HRAS*, *IDH1*, *IDH2*, *JAK2*, *JAK3*, *KDR*, *KIT*, *KRAS*, *MAP2K1*, *MET*, *MLH1*, *MPL*, *MSH6*, *NOTCH1*, *NPM1*, *NRAS*, *PDGFRA*, *PIK3CA*, *PTEN*, *PTPN11*, *RB1*, *RET*, *SMAD4*, *SMARCB1*, *SMO*, *SRC*, *STK11*, *TP53*, *VHL*.

This control was calibrated using the analysis parameters detailed in the CLIA validation. The resulting analysis yielded 351 detected variants, and these variants served as the reference set for quality control of each sequencing run. As a quality control measure for variant detection, a minimum of 344 variants (95%) must be identified from each sequencing run for variants from clinical specimens to be reported. A detailed quality control log was maintained, which documented the results from each run and was a part of the routine CLIA compliance.

### 2.4. Data Analysis

All statistical analyses were performed using JMP^®^ software by the SAS Institute (Cary, NC, USA). The distributions of the serum galectin levels were analyzed for normality. The distributions for each galectin in a mutated gene were compared to those of patients with a non-mutated version of the same gene by *t*-test. 

Contingency analyses were performed on the mutation status of genes against other categorical variables, such as tissue site and histology. The odds ratios were calculated for the *KIT* mutations and brain metastases in both cancers. Both cancers were analyzed separately. Values of *p* less than 0.05 were considered statistically significant.

## 3. Results

The levels of circulating galectins -1, -3, -7, -8 and -9 in breast and lung cancer patients were revealed by an ELISA assay of patient serum [65]. Tumor tissues from the same patients were analyzed for mutations in 50 cancer-critical genes by multiplex polymerase chain reaction (PCR). The mutation status of these genes was compared to the circulating levels of galectins in the cancer patients.

### 3.1. Serum Galectin Levels

Table 1 and Table 2 contain the serum galectin levels for the cancer patient groups. Some samples were excluded from further analysis due to the reliability of the results.

### 3.2. Mutation Frequency

Table 3 ranks the genes by frequency of mutation among breast cancer patients as well as the specific mutation. *PIK3CA* and *TP53* were the most mutated genes in this group patients. 

Table 4 provides the mutations and their frequencies in the lung cancer patient sample group. *TP53* and *KDR* (VEGF2) were the most mutated genes in this group.

### 3.3. Associations with Galectins

**Figure 2:**
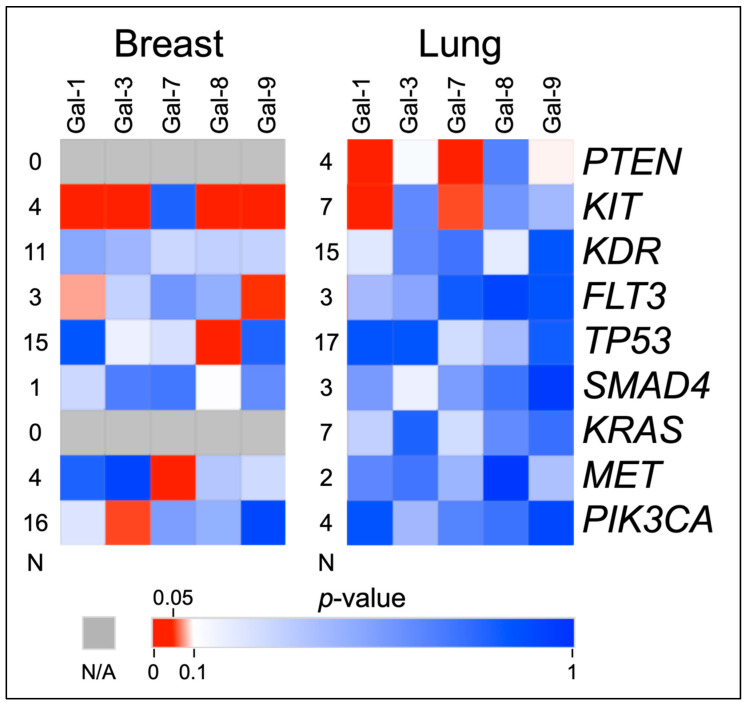
Heat Map of Mutated versus Wild-Type Galectin Levels T-Tests. This figure shows the *p*-values of t-test comparisons of the serum galectin levels in mutated vs. non-mutated genes in breast and lung cancer patients. Levels of galectin-1, -3, -8, and -9 are significantly elevated when *KIT* is mutated in breast cancer samples. Levels of galectin-1 and -7 are significantly elevated when *PTEN* and *KIT* are mutated in lung cancer samples. “N” indicates the number of mutated samples in the comparison.

The screening of galectin levels by gene mutations found several associations between the serum galectin levels and cancer-critical gene mutations. Most notable are the associations with multiple galectin levels and the *KIT* gene. Figure 2 shows a heat map of the t-test results of comparing the serum galectin levels in patients with a mutated gene to patients with a wild-type gene. 

**Figure 3:**
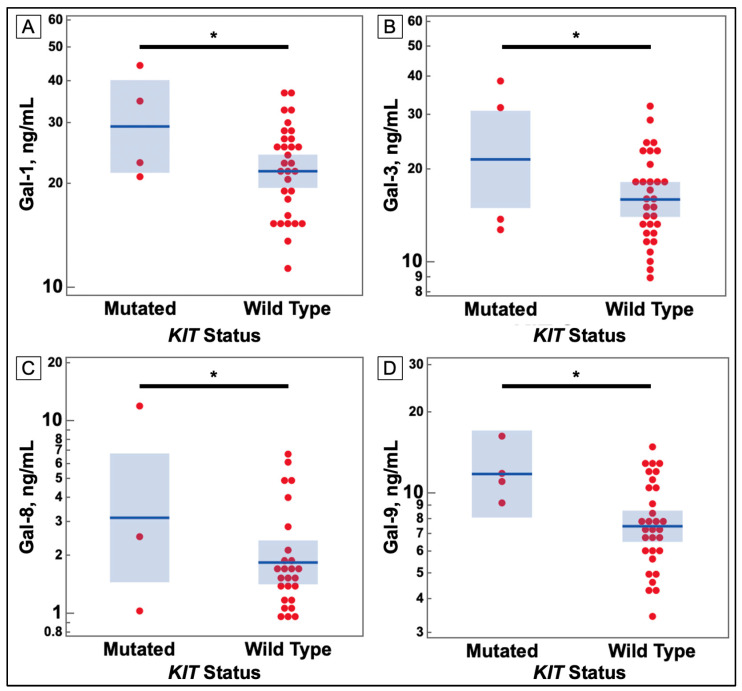
Comparison of Serum Galectin Levels of Breast Cancer Patients *KIT* Gene Mutation Status in Tumor Sample. (**A**) Serum levels of galectin-1, as determined by ELISA, were significantly higher in breast cancer patients with a mutation in the *KIT* gene. (**B**) Serum levels of galectin-3, as determined by ELISA, were significantly higher in breast cancer patients with a mutation in the *KIT* gene. (**C**) Serum levels of galectin-8, as determined by ELISA, were significantly higher in breast cancer patients with a mutation in the *KIT* gene. (**D**) Serum levels of galectin-9, as determined by ELISA, were significantly higher in breast cancer patients with a mutation in the *KIT* gene. (* *p*-value ≤ 0.05).

**Figure 4:**
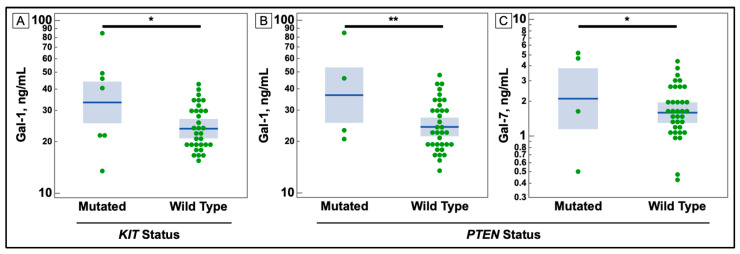
Comparison of Serum Galectin Levels of Non-small Cell Lung Cancer Patients with *KIT* and *PTEN* Mutations in Tumor Sample. (**A**) Serum levels of galectin-1, as determined by ELISA, were significantly higher in non-small cell lung cancer patients with a mutation in the *KIT* gene. (**B**,**C**) Serum levels of galectins -1, and -7 were elevated in patients with a mutation in the *PTEN* gene. (* *p*-value ≤ 0.05, ** ≤ 0.01).

Figure 2 shows that despite the small number of mutations in the *KIT* gene, they correlated with increases in several galectins in both cancers. These comparisons are charted in Figure 3 and Figure 4. Additionally, *PTEN* was found to have some correlations with galectin levels in NSCLC patients, as seen in Figure 4. 

### 3.4. Associations with Brain Metastases

**Figure 5:**
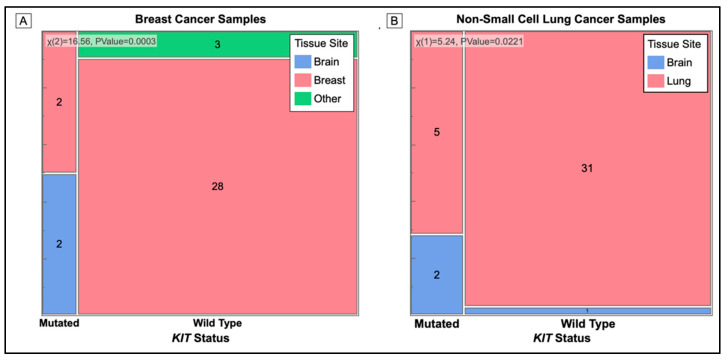
Mosaic Plot of Cancer Tissue Biopsy Site (Primary and Metastatic) used for Mutation Analysis. Cell sizes are proportional to the number of samples in that category. Mutation status is on the X-axis and color represents the tissue sample of origin. (**A**) All *KIT* mutations occur in biopsy samples of breast cancer taken from the brain. The *p*-value = 0.0003. (**B**) Biopsy samples taken from the brain have a higher number of *KIT* mutations than samples from the lung. The *p*-value = 0.0221.

Figure 5 shows the contingency analysis of the presence of a *KIT* mutation at the site of the tissue biopsy of the tumor. Tumor samples taken from the brains of cancer patients were significantly more likely to have a mutation in the *KIT* gene.

Table 5 shows the odds ratio between having a *KIT* mutation and brain metastasis. Our sample population did not contain a breast cancer sample with a brain metastasis and wild-type *KIT* and therefore, no ratio could be calculated for the group.

In summary, in breast cancer patients, we find that *PIK3CA* and *TP53* were the most mutated genes while *TP53* and *KDR* (VEGF2) were the most mutated genes in the lung cancer patients. Levels of galectins -1, -3, -8, and -9 were elevated in patients with mutations in the *KIT* gene. Simultaneously, samples from a brain metastasis of breast and lung cancer patients had more *KIT* gene mutations than samples from the primary tumor.

## 4. Discussion

Galectins -1, -3, -8, and -9 were found to be at higher levels in sera of breast cancer patients with a mutation in the *KIT* gene than other cancer patients without the mutation. 

The most common *KIT* mutation in our panel was p.Met541Leu (rs3822214). This mutation occurs in the transmembrane region of the protein and has not been implicated as a mutation of clinical concern [98]. Since the mutation occurs in the transmembrane region, some have theorized that the mutation is loss-of-function and impairs the insertion of the receptor into the membrane [99]. However, studies have shown that the p.Met541Leu mutation increases the RTK’s affinity for its ligand, SCF [100,101]. One study found that chronic myelogenous leukemia (CML) patients with this mutation had altered white blood cell counts and overall survival [101].

Our study joins others in finding increasing potential clinical significance for this missense mutation [102]. We investigated the allele frequency of the mutation in these patients and found it indicates a heterozygous germline mutation. This is supported by studies which find that this mutation is common (8.1% allele frequency) in the Caucasian population [103]. For comparison, the mutation appears in 8.57% of our breast cancer patients and 15.38% of the lung cancer patients for 12.61% overall.

Galectins and RTKs, such as c-Kit, are known to have an abundant number of interactions [104]. There is no literature to indicate specific interactions between galectins -1, -3, -8, and -9 and c-Kit, although it is known that galectins do interact with other members of this class of RTKs, such as platelet-derived growth factor receptor (PDGFR) via spatial organization and trafficking [105,106,107].

**Figure 6:**
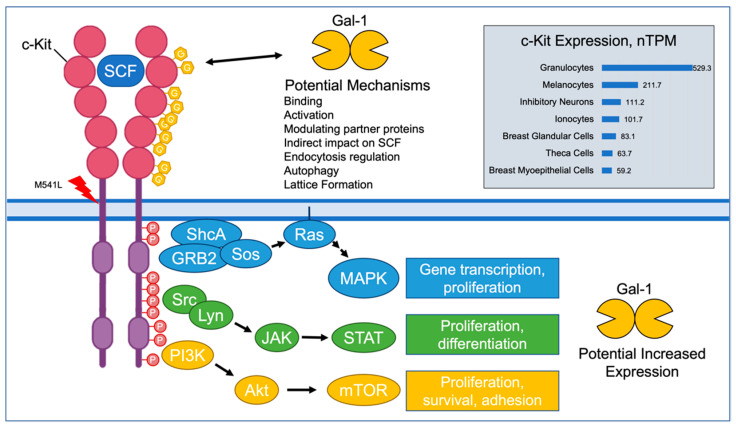
Potential Interactions between c-Kit and Galectin-1. c-Kit is expressed in immune cells, melanocytes, neurons, and breast tissue cells. Galectin-1 can bind to appropriate glycosylation sites on c-Kit with a variety of possible results affecting c-Kit functioning. The downstream effects of increased c-Kit activity could include increased transcription of galectin-1 levels. (SCF: stem cell factor; nTPM: normalized transcriptions per million; Gal-1: galectin-1) [10,104,108,109].

The association between the c-Kit mutation and increased levels of certain galectins is interesting. There are a variety of possible interpretations of this finding (Figure 6). The mutation could lead to altered receptor glycosylation, which would in turn affect the galectin serum levels. Galectin expression could be upregulated by the GOF c-Kit mutations via the activated intracellular pathways. The nature of the interaction is of interest and worthy of future studies.

A query of the TCGA database via UALCAN shows that galectins -1, and -3, have decreased expression in breast invasive carcinoma, suggesting that the observed increase in these galectins could be of a non-tumor origin. The database also shows that galectins -8 and -9 have increased expression in breast cancer tissue [110].

This study also found that tissue samples taken from the metastasis in patients’ brains were more likely to have a mutated *KIT* gene. It is unclear why a mutated c-Kit protein would result in this outcome and, in fact, one study has shown that the *loss* of c-Kit expression has been associated with advanced stages of breast cancer [111]. It is possible that the mutation reduces the stability of the c-Kit protein.

### 4.1. Impact of Findings

These findings serve to further enhance the understanding of the role of galectins in the cancer setting. Serum levels of certain galectins are known to be increased in cancer [65]. Our study shows that certain galectins could have increased serum levels when certain cancer-critical genes are mutated in the tumor sample, indicating a potential relationship.

Additionally, given the high frequency of the p.Met541Leu c-Kit mutation in the general population, its cause for concern in other studies [101,102] and its correlation with brain metastasis in cancer patients of this study, the p.Met541Leu mutation is a potential marker for more aggressive cancer and has promise for future studies.

The practical application of this research is the discovery of further molecular changes correlated with specific tumor mutations. The Ampliseq hotspot panel provides a gene panel that can be used to investigate many genes of interest, not only in breast and lung cancers but in other cancers and diseases as well.

Further investigations could find blood serum markers that better correlate with the mutation status of cancer-critical genes. This approach has applications in both diagnostics and treatment, as the mutation status of specific proteins often translates to their response to cancer treatments. For example, p.Met541Leu KIT-expressing cells have been shown to have increased sensitivity to imatinib, a c-Kit inhibitor [112]. This is a practical goal, as cancer treatment is tailored to specific mutations and the galectin levels can be targeted by galectin inhibitors.

### 4.2. Study Limitations

Our sample size of 35 breast cancer samples and 39 lung cancer samples reflects the availability of the hotspot panel sequencing data and the pilot nature of this study. Due to the method of sample selection, a traditional power calculation was not performed. The size of the sampling does limit the generalizability of the study. However, we view this work as an exploratory study and a way to find and flag potential genes and gene mutations of interest.

Additionally, we did not control for other patient variables, such as comorbidities and detailed treatment, due to the boundaries of our approved research scope. In regard to the treatment information, our previous work found that the galectin levels in treated versus untreated or not recently treated for this sample group had no observable differences [65]. As such, our study should be interpreted accordingly, as a heterogeneous pool of cancer patients representing the population from which they were obtained.

## 5. Conclusions

Based on our findings, we propose areas for future studies. The first is a mechanistic analysis of potential binding between galectins and glycosylated c-Kit protein. Second, is the establishment of the role of c-Kit in the regulation of expression and secretion of galectins. Third, is the investigation into the relationship between mutated c-Kit proteins and metastatic brain tumors. Further, c-KIT and its ligand, SCF, are known to be expressed preferentially in small cell lung cancers [113]. As small cell lung cancers were not examined in this study, the next step would be to examine the c-Kit mutation status and galectin levels in SCLC to determine if there is a correlation. Finally, the concept of a hotspot gene panel to find correlations between the mutations and circulating biochemical markers can be expanded to cover more cancer types and molecular markers. As a result, these studies should not only provide new insight into the key aspects of c-Kit and galectin interactions but may also provide an important framework to create rational approaches to prevent the development of metastasis in other cancers.

## Figures and Tables

**Table 1:** Breast Cancer Patient Serum Galectin Levels.

Galectin	*n*	Mean, ng/mL	SD, ng/mL	Min-Max, ng/mL
Gal-1	35	23.50	7.48	11.30–43.92
Gal-3	35	17.52	6.94	8.85–38.20
Gal-7	35	1.77	1.85	0.58–11.69
Gal-8	29	2.50	2.35	0.95–11.84
Gal-9	35	8.40	3.21	3.42–16.12

**Table 2:** Lung Cancer Patient Serum Galectin Levels.

Galectin	*n*	Mean, ng/mL	SD, ng/mL	Min-Max, ng/mL
Gal-1	39	27.12	12.84	13.41–83.69
Gal-3	38	17.77	6.98	4.52–37.82
Gal-7	39	1.91	1.12	0.42–5.07
Gal-8	31	2.73	8.02	0.56–45.71
Gal-9	39	10.15	4.87	3.79–26.64

**Table 3:** Gene Mutation Frequency in Breast Cancer Patients.

Gene	Mutation	Count	Percent
*PIK3CA*	p.His1047Arg	7	20.0%
	p.Ile391Met	5	14.3%
	p.Glu545Lys	4	10.3%
	Other	2	5.7%
	Total	18	51.4%
*TP53*	p.Pro72Arg	5	14.3%
	p.Arg273His	2	5.7%
	Other	10	28.6%
	Total	17	48.6%
*KDR*	p.Gln472His	11	31.4%
	Total	11	31.4%
*KIT*	p.Met541Leu	3	8.6%
	p.Val530Ile	1	2.9%
	Total	4	11.4%
*MET*	p.Asn375Ser	2	5.7%
	p.Met362Thr	2	5.7%
	Total	4	11.4%
Other	Other	14	40.0%

**Table 4:** Gene Mutation Frequency in Lung Cancer Patients.

Gene	Mutation	Count	Percent
*TP53*	p.Pro72Arg	4	10.2%
	Other	14	35.9%
	Total	18	46.2%
*KDR*	p.Gln472His	15	38.5%
	p.Val1356Ala	1	2.6%
	Total	16	41.0%
*KIT*	p.Met541Leu	6	15.4%
	p.Glu849Gln	1	2.6%
	Total	7	18.0%
*KRAS*	p.Gly12Asp	2	5.1%
	p.Gly12Cys	2	5.1%
	p.Gly13Cys	2	5.1%
	p.Gly13Asp	1	2.6%
	Total	7	18.0%
*PIK3CA*	p.Ile391Met	3	7.7%
	p.His1047Arg	1	2.6%
	Total	4	10.3%
*PTEN*	p.Arg173fs	1	2.6%
	p.Gly165Ter	1	2.6%
	p.Gly244Cys	1	2.6%
	p.Met1Ile	1	2.6%
	Total	4	10.3%
Other	Other	29	74.4%

**Table 5:** Odds Ratio of *KIT* Mutation and Brain Metastasis in Breast and Lung Cancer Patients.

Cancer	Mutated *KIT*		Wild-Type *KIT*		
	Brain Met	No Brain Met	Brain Met	No Brain Met	Odds Ratio
Breast	2	2	0	31	-
Lung	2	5	1	31	12.4
Total	4	7	1	62	35.4

## Data Availability

The data presented in this study is available in this article.

## Supplementary Materials

The following supporting information can be downloaded at: https://www.mdpi.com/article/10.3390/cancers14112781/s1, Table S1: Breast Cancer Patient Sample Statistics; Table S2: Lung Cancer Patient Sample Statistics; Table S3: ELISA quality control measurements.

## Abbreviations

PCR, Polymerase Chain Reaction; ELISA, Enzyme-Linked Immunosorbent Assay; HER2, Human Epidermal Growth Factor Receptor 2; NSCLC, Non-Small Cell Lung Cancer; EGFR, Epidermal Growth Factor Receptor; ALK, Anaplastic Lymphoma Kinase; RTK, Receptor Tyrosine Kinase; RAS, Rat Sarcoma; MAP, Mitogen Activated Protein; TGFβ, Transforming Growth Factor Beta; PI3K, Phosphoinositide 3-kinase; Wnt, Wingless-Related Integration Site; GPCR, G-Protein Coupled Receptor; p53, Cellular Tumor Antigen p53; JAK, Janus Kinase; STAT, Signal Transducer and Activator of Transcription Proteins; Tim-3, T-cell Immunoglobulin Mucin 3; GR-MD-02, Belapectin; TNM, Tumor Node Metastasis; MMP-2, Matrix Metalloproteinase 2; MMP-9, Matrix Metalloproteinase 9; Cdc42, Cell Division Control Protein 42; Akt, Protein Kinase B; PHCI, Prisma Health Cancer Institute; ABL1, Tyrosine-Protein Kinase ABL1; Akt1, RAC-alpha Serine/Threonine Protein Kinase; BRAF, Serine/Threonine Protein Kinase BRAF; CSF1R, Colony Stimulating Factor 1 Receptor; CTNNB1, Catenin-beta 1; ERBB2, Human Epidermal Growth Factor Receptor 2; ERBB4, Receptor Tyrosine-Protein Kinase ERBB4; EZH2, Enhancer of Zeste Homolog 2; FGFR1, Fibroblast Growth Factor Receptor 1; FGFR2, Fibroblast Growth Factor Receptor 2; FGFR3, Fibroblast Growth Factor Receptor 3; FLT3, Fetal Liver Tyrosine Kinase 3; GNA11, Guanine Nucleotide-Binding Protein Subunit Alpha 11; GNAQ, Guanine Nucleotide-Binding Protein Subunit Alpha; GNAS, Guanine Nucleotide-Binding Protein Alpha Stimulating; HRAS, GTPase HRAS; IDH1, Isocitrate Dehydrogenase 1; IDH2, Isocitrate Dehydrogenase 2; JAK2, Tyrosine-Protein Kinase JAK2; JAK3, Tyrosine-Protein Kinase JAK3; KDR, Vascular Endothelial Growth Factor Receptor 2; KIT, Mast/Stem Cell Growth Factor Receptor KIT; KRAS, GTPase KRAS; MET, Hepatocyte Growth Factor Receptor; MPL, Thrombopoietin Receptor; NPM1, Nucleophosmin 1; NRAS, GTPase NRAS; PDGFRA, Platelet-Derived Growth Factor Receptor A; PIK3CA, Phosphatidylinositol 4;5-Bisphosphate 3-Kinase Catalytic Subunit Alpha Isoform; PTPN11, Tyrosine-Protein Phosphatase Non-Receptor Type 11; RET, Proto-Oncogene Tyrosine-Protein Kinase Receptor RET; SMARCB1, SWI/SNF-Related Matrix-Associated Actin-Dependent Regulator of Chromatin Subfamily B Member 1; SMO, Smoothened; SRC, Proto-Oncogene Tyrosine-Protein Kinase SRC; APC, Adenomatous Polyposis Coli Protein; ATM, Ataxia Telangiectasia Mutated; CDH1, Cadherin 1; CDKN2A, Cyclin Dependent Kinase Inhibitor 2A; FBXW7, F-box WD Repeat-Containing Protein 7; HNF1A, Hepatocyte Nuclear Factor 1 Homeobox A; MLH1, MutL Homolog 1; NOTCH1, Neurogenic Locus Notch Homolog 1; PTEN, Phosphatase and Tensin Homolog; RB1, RB Transcriptional Corepressor 1; SMAD4, Mothers Against Decapentaplegic Homolog 4; STK11, Serine/Threonine Kinase 11; TP53, EKC/KEOPS Complex Subunit TP53RK; VHL, Von Hippel Lindau Disease Tumor Suppressor; SCF, Stem Cell Factor; GIST, Gastrointestinal Stromal Tumors; CML, Chronic Myelogenous Leukemia; nTPM, Normalized Transcriptions per Million; mRNA, Messenger Ribonucleic Acid; Gal-1, Galectin-1; Gal-3, Galectin-3; Gal-7, Galectin-7; Gal-8, Galectin-8; Gal-9, Galectin-9; GOF, Gain of Function; TCGA, The Cancer Genome Atlas; UALCAN, The University of Alabama at Birmingham Cancer Data Analysis Portal; SCLC, Small Cell Lung Cancer.
