# Supplementary material for: KIT Mutations Correlate with Higher Galectin Levels and Brain Metastasis in Breast and Non-Small Cell Lung Cancer
Source: Cancers (Basel). 2022 Jun 3;14(11):2781. doi: 10.3390/cancers14112781 (PMC9179545; doi:10.3390/cancers14112781)
Supplement: Supplementary file 1 [file cancers-14-02781-s001.zip › Supplemental.pdf]

Supplemental Materials

Table S1. Breast Cancer Patient Sample Statistics

Characteristic	N	% of Total
**Gender**		
Female	35	100.00%
**Race**		
Black, African American	7	20.00%
Latino	1	2.86%
White	27	77.14%
**Ethnicity**		
Non-Spanish; Non-Hispanic	34	97.14%
Spanish; Hispanic	1	2.86%
**Smoking History**		
Current smoker	3	8.57%
Never smoked	19	54.29%
Previous smoker	13	37.14%
**T**		
T1a	0	0.00%
T1b	4	11.43%
T1c	7	20.00%
T2	13	37.14%
T3	4	11.43%
T4	1	2.86%
T4a	1	2.86%
**N**		
N0	18	51.43%
N1	3	8.57%
N1a	2	5.71%
N2a	4	11.43%
N3	2	5.71%
**M**		
M0	16	45.71%
M1	1	2.86%
**Stage**		
I	10	28.57%
II	10	28.57%
III	10	28.57%
IV	5	14.29%
**Tissue Site**		
Brain	2	5.71%
Breast	30	85.71%
Liver	1	2.86%
Lymph Node	1	2.86%
Ovary	1	2.86%
**Sample Considered**		
Metastatic	4	11.43%
Primary	29	82.86%
Recurrence	2	5.71%
**Histology**		
Adenocarcinoma	2	5.71%

Ductal	31	88.57%
Lobular	2	5.71%
**Grade**		
1	5	14.29%
2	13	37.14%
3	13	37.14%
U	4	11.43%

Table S2. Lung Cancer Patient Sample Statistics

Characteristic	N	% of Total
**Gender**		
Female	17	43.59%
Male	22	56.41%
**Race**		
Black, African American	9	23.08%
Latino	1	2.56%
White	29	74.36%
**Ethnicity**		
Non-Spanish; Non-Hispanic	38	97.44%
Spanish; Hispanic	1	2.56%
**Smoking History**		
Current smoker	12	30.77%
Never smoked	3	7.69%
Previous smoker	24	61.54%
**T**		
T1	1	2.56%
T1a	2	5.13%
T1b	4	10.26%
T1c	1	2.56%
T2	1	2.56%
T2a	7	17.95%
T2b	4	10.26%
T3	7	17.95%
T4	1	2.56%
**N**		
N0	13	33.33%
N1	11	28.21%
N2	3	7.69%
**M**		
M0	10	25.64%
M1	4	10.26%
**Stage**		
I	10	25.64%
II	10	25.64%
III	10	25.64%
IV	9	23.08%
**Tissue Site**		
Brain	3	7.69%
Lung	36	92.31%

**Sample Considered**		
Metastatic	3	7.69%
Primary	36	92.31%
**Histology**		
Adenocarcinoma	24	61.54%
Large Cell	2	5.13%
Squamous Cell	13	33.33%

No information was available for Grade for these patients.

Table S3. ELISA quality control measurements

Galectin	Catalog No.	Company	Mean Intra-Assay Precision, CV%	Mean Inter-Assay Precision, CV%	Minimum Detectable Dose, ng/mL
Gal-1	DGAL10	R&D Systems	7.2	8.6	0.022
Gal-3	DGAL30	R&D Systems	3.7	7.8	0.016
Gal-7	EHLGALS7	Invitrogen	<10%	<12%	0.020
Gal-8	EH205RB	Invitrogen	<10%	<12%	0.16
Gal-9	DGAL90	R&D Systems	3.6	5.4	0.008

For further assay specifications, please refer to the manufacturer documentation.
